# Vitamin K Intake in Chronic Stroke: Implications for Dietary Recommendations

**DOI:** 10.3390/nu12103059

**Published:** 2020-10-06

**Authors:** Chad Wessinger, Charlene Hafer-Macko, Alice S. Ryan

**Affiliations:** VA Research Service, Department of Medicine, Division of Gerontology and Geriatric Medicine at the University of Maryland School of Medicine, and the Baltimore VA Medical Center Geriatric Research, Education and Clinical Center (GRECC), VA Maryland Health Care System, Baltimore, MD 21201, USA; cwessinger@som.umaryland.edu (C.W.); chafer-macko@som.umaryland.edu (C.H.-M.)

**Keywords:** vitamin K, stroke, chronic stroke, food records

## Abstract

Previous research has identified a possible association between vitamin K intake and cardiometabolic disease. This could mean that the assessment of vitamin K intake is a meaningful tool when monitoring individuals with preexisting cardiovascular disease. Sixty chronic stroke survivors (men and women, body mass index (BMI) 30.36 ± 6.61 kg/m^2^, age 61.7 ± 7.2 years) completed food records which were analyzed for energy, macronutrient, micronutrient, and food group servings. Participants were divided into two groups: below vitamin K recommendation (BEL, *n* = 49) and met vitamin K recommendation (MET, *n* = 11). Energy and macronutrient intake did not differ between groups (all *p* > 0.127). Vegetable intake was higher in the MET group (*p* = 0.0001). Vitamin K intake was higher in the MET group (*p* = 0.0001). Calcium (*p* = 0.003), vitamin A (*p* = 0.007), and vitamin E (*p* = 0.005) intakes were higher in the MET group. There were no differences in sodium, potassium, vitamin D, vitamin C, and iron intakes between groups (all *p* > 0.212). In this sample of chronic stroke survivors, 82% reported consuming below the Dietary Reference Intake (DRI) for vitamin K. Given that the majority of this study population did not reach the DRI for vitamin K, it is advisable to promote the adequate intake of food rich in vitamin K. Further work is needed to determine the significance of low vitamin K intake in this population.

## 1. Introduction

The assessment of macronutrient and fatty acid intake is an effective method to identify individuals at increased risk of the development of cardiometabolic disease [1]. In addition to the assessment of macronutrient and fatty acid intake, micronutrient intake is also important for identifying dietary deficiencies [2]. This is particularly true for the micronutrient intake of vitamin K, as previous work indicates a potential association between low vitamin K intake and cardiometabolic disorders [3,4]. Vitamin K intake mirrors the intake of leafy green vegetables [5], and a higher or at least an adequate consumption of fruits and vegetables is associated with a decreased risk of the development of cardiovascular disease [6]. Although a causal relationship has not been established between vitamin K and cardiometabolic disorders, their close association with fruit and vegetable intake could provide another measure related to cardiovascular disease.

There are several therapeutic implications of vitamin K intake. One of vitamin K’s main functions is as a coenzyme for vitamin K-dependent carboxylase, an enzyme responsible for the synthesis of prothrombin, matrix Gla-protein, and osteocalcin. Prothrombin, also known as clotting factor II, is directly involved in blood clotting [7]. It is thought that drug regimens of antithrombotic medications combined with a consistent intake of vitamin K could reduce the incidence of bleeding and thrombotic complications [8,9]. Vitamin K administration is also a key part of the treatment strategy to reverse an unstable international normalized ratio (INR) [10]. Additionally, one paper has identified the possibility of an association between a genetic predisposition for higher serum levels of vitamin K and a higher incidence of stroke [11]. However, there is also evidence to suggest that higher intake of vitamin K (especially in the form of menoquinones MK-7, MK-8, and MK-9) may protect against the development of coronary heart disease [12]. Vitamin K supplementation has also been shown to be beneficial in combating vascular calcification [13]. Deficiency of vitamin K is related to the development of interstitial pneumonias, which may be corrected, in part, via controlled vitamin K supplementation [14]. For these reasons, vitamin K may be a key target to identify those at risk of bleeding and thrombotic complications when conducting a dietary assessment.

Because cardiovascular disease risk factors can be ameliorated with improved diet quality [6], dietary assessment may be an important tool to monitor in stroke survivors given their prior thrombotic event. It is common practice to prescribe antithrombotic drugs to patients who have survived a stroke to prevent further cardiovascular events [15]. The potential relationship between vitamin K intake and cardiometabolic disease [3,4] introduces vitamin K intake as a conceivable additional measure when assessing chronic stroke survivors’ risk of recurrence.

The aim of this study was to determine the frequency of adequate or inadequate intake of vitamin K and to compare micronutrients, macronutrients, food group servings, and medications between adults with chronic stroke who met (MET) or did not meet (BEL) the Dietary Reference Intakes (DRI) for vitamin K.

## 2. Materials and Methods

### 2.1. Participants

Stroke survivors (*n* = 60) with residual hemiparetic deficits > six months after the onset of ischemic stroke participated in this study. Participants were recruited through local advertisements, flyers and stroke clinics at the University of Maryland School of Medicine and Baltimore VA Medical Center. All participants with stroke lived independently in the community and were able to walk six minutes with their usual assistive device(s) without human assistance. Evaluations included medical history, physical examination, fasting blood profile, and screening for dementia and depression to ensure adequate informed consent. Participants with stroke were excluded if they had unstable angina, congestive heart failure (NYHA II), severe peripheral arterial disease, end-stage organ disease, major post-stroke depression, dementia, severe receptive aphasia, orthopedic or chronic pain conditions. The Institutional Review Board of the University of Maryland and the Baltimore VA Research & Development Committee approved all methods and procedures. Each participant provided written informed consent.

### 2.2. Nutrition

Participants visited with a Registered Dietitian (RD) and were instructed on how to complete a food record. Each participant completed a 3 to 7-day food record by recording all foods consumed and the estimated serving size of each food. Food records were analyzed for energy and macronutrient intake and distribution. An analysis of micronutrient intake, including sodium, potassium, vitamin K, vitamin D, calcium, vitamin C, vitamin A, iron, and vitamin E was also performed. Exchange list serving sizes were assessed for each day. The average of the daily records for each index was used. The analysis of the food records was done using Nutritionist Pro software. Participant intake was compared to the Dietary Reference Intakes (DRIs) [16,17].

### 2.3. Body Composition and Physical Function

Height (cm) and weight (kg) were measured to calculate BMI. Percent body fat mass was determined by dual-energy X-ray absorptiometry (iDXA, LUNAR Radiation Corp, Madison, WI). Participants underwent a six-minute walk test (6MWD), which measures ambulation distance and represents what may be required for community-based activities of daily living (ADL). This test has been shown to be an effective measure of gait endurance [14]. Participants were instructed to “cover as much distance as possible” over a flat 100-foot surface marked by traffic cones during a 6-min time period. Assistive devices were used by participants when necessary. On a separate day, participants completed a 10-m walk test used to gauge walking speed over a shorter distance. Both self-selected or usual walking speed (10MUS) and fastest comfortable walking speed (10MFS) were assessed. Standard commands and instructions were used during these walking assessments. In addition, hand grip strength was measured using a hydraulic hand grip dynamometer (model 12-0240, Fabrication Enterprises, Inc., Warwick, RI, USA). Both arms were used for testing while bent at a right angle with the elbow at the participants’ side. Participants were instructed to squeeze the dynamometer handle with maximal effort for five seconds three separate times, with the best of the three used for statistical analyses. Due to scheduling conflicts for participants, data are missing for DXA (*n* = 7), walking distance (*n* = 7), gait speed (*n* = 14), and grip strength (*n* = 14).

### 2.4. Statistical Analyses

Descriptive statistics and comparisons between groups were analyzed using unpaired t-tests with SPSS (PASW Statistics, Version 22, Chicago, IL, USA). Relationships between variables were determined by linear regression analyses with the calculation of Pearson product moment correlation coefficients. Data are presented as the mean ± SD. Statistical significance was set at a two-tailed *p*-value < 0.05.

## 3. Results

### 3.1. Demographic Characteristics

Sixty chronic stroke survivors (*n* = 49 men, *n* = 11 women) aged 54 to 68 years completed the study (Table 1). Approximately 56% of participants were African American and the remainder were Caucasian. On average, participants completed 4.6 ± 1.7 days of a dietary food record. As a whole, and in men only, participants were obese (class one), whereas women fell into the overweight classification. DXA and physical function measures, as presented in Table 1, showed reduced gait speed and mobility. All (100%) of the participants were on at least one medication with 42 (70%) of the individuals on an antiplatelet medication, 39 (65%) on statin therapy, and 45 (75%) on antihypertensive medications. Only six participants were on anticoagulants (three on warfarin), four on insulin (7%) and 20 on oral agents for diabetes (33%), and 18 on a diuretics (30%). No participants were on antibiotics at the time of the study.

### 3.2. Dietary Intake

The dietary intake of the total group and divided into men and women is shown in Table 2. Participants consumed 17% of their intake from protein and 47% and 35% of their energy needs from carbohydrate and fat, respectively. Sodium intake was high and, on average, servings of fruit and vegetables were low.

Of the 60 participants in this study, 49 (82%, *n* = 41 men and *n* = 8 women) consumed under the DRI for vitamin K and 11 (18%, n = 8 men and n = 3 women) met or exceeded the DRI for vitamin K. When the total group was divided by those who MET and were BEL vitamin K intake, the mean age (59.6 ± 7.5 vs. 62.5 ± 7.2 years, *P* = 0.89) and BMI (33.21 ± 5.87 vs. 30.24 ± 6.68 kg/m^2^, *P* = 0.87) was not different between groups. In addition, percent body fat was not different between groups (MET vs. BEL: 38.1 ± 5.2 vs. 34.6 ± 8.2%). Likewise, fat mass (38.5 ± 8.8 vs. 32.0 ± 12.5 kg) and lean mass (56.60 ± 12.9 vs. 55.1 ± 9.9 kg) did not differ between MET and BEL groups, respectively. There were no differences in the measures of physical function (6-min walk feet, 10-m walk usual and fast pace, grip strength) between the two groups (data not shown).

### 3.3. Macronutrient Intake and Food Group Exchanges by Vitamin K Intake

There was no difference between the BEL group and the MET group in energy or macronutrient intake. The intake of starch, fruit, fat, high fat meat, medium fat meat, lean meat, very lean meat, and other carbohydrate exchange equivalents were also not different between groups. However, those who met the DRI for vitamin K had a higher intake of vegetables (*p* = 0.0001) and skim milk (*p* = 0.007) exchanges (Table 3).

### 3.4. Micronutrient Intake

**Figure 1:**
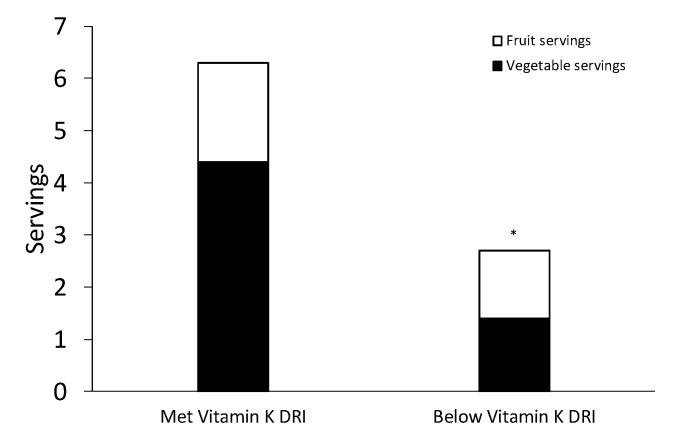
Total combined fruit and vegetable servings per day. *p = 0.05. DRI—Dietary Reference Intake.

Those who met the DRI for vitamin K had higher intakes of calcium (*p* = 0.003), vitamin A (*p* = 0.007), and vitamin E (*p* = 0.005) than those whose intake of vitamin K was below the DRI. There were no differences in the intake of sodium, potassium, vitamin D, vitamin C, and iron between the two groups (Table 4). Servings of fruits and vegetables were lower in those who were BEL vitamin K intake recommendations than those who MET recommendations (*p* = 0.05, Figure 1). Dietary intake of vitamin K was associated with dietary intake of vitamin A (r = 0.49, *p* = 0.0001) and calcium (r = 0.30, *p* = 0.021).

**Figure 2:**
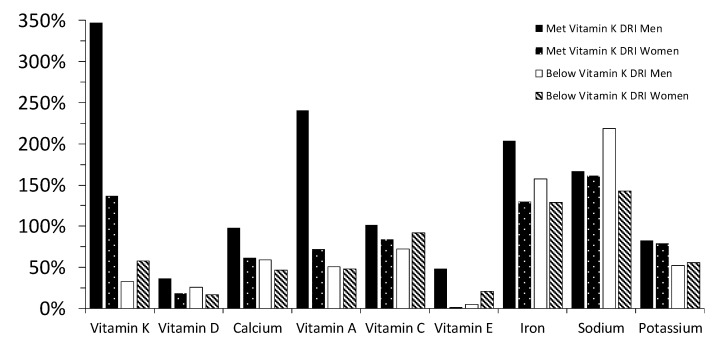
Micronutrient intake expressed as percentage of DRI.

The micronutrient intake of other vitamins and minerals in relation to the level of vitamin K intake for men and women separately is presented in Figure 2. On average, men and women in the MET group reached 348% and 137% of the DRI for vitamin K, respectively, while for those in the BEL intake for vitamin K group, men and women reached only 33% and 58% of the DRI for vitamin K, respectively.

Participants in all groups were below the DRI for vitamin D and similar results were found among those who MET and were BEL for both men (37 vs. 25%) and women (18 vs. 17%), respectively. Men in the MET group reached 98% of the DRI for calcium. However, women in the MET group reached only 62% of the DRI for calcium. The percent of DRI intake for calcium was similar for men and women in the BEL group (59% and 46%, respectively). For vitamin A, men reached 241% and 51%, whereas women reached 72% and 48% of the vitamin A DRI, in the MET and BEL, groups respectively. In the MET and BEL groups, men’s vitamin C intake was 101% vs. 72% and women reached 84% and 92% for vitamin C. The reported vitamin E intake was below DRI among all groups (48%, 1%, 5%, and 21% of the DRI for men in the MET group, women in the MET group, men in the BEL group, and women in the BEL group, respectively). Reported iron intake was higher than DRI in all groups ranging between 129 and 204%. Sodium intake was also above the DRI for men and women in the MET (167% and 218%) and BEL groups (161% and 143%). Potassium intake was close to the recommended intake in the MET group for men and women (82% and 79%) but approximately half the recommended intake in both BEL groups (men at 52% and women at 56%).

### 3.5. Dietary Supplements

Only 18% of participants reported use of a multivitamin supplement, while the other 82% of participants did not report the use of such a supplement. Most participants that reported no use of a multivitamin supplement did not meet the DRI for vitamin K. All participants that reported no use of a vitamin D supplement did not reach the DRI for vitamin D. Most participants reported no calcium supplementation. The majority of participants that did not use a calcium supplement did not reach the DRI. Of the 49 participants that reported no multivitamin supplementation, more than half did not reach the DRI for vitamin A or vitamin C. Of the 49 participants that reported no multivitamin use, most did not reach the DRI for vitamin E (Table 5).

## 4. Discussion and Conclusions

Our results indicate that the majority of stroke participants have a low dietary vitamin K intake regardless of age and sex. This is related to a low intake of fruits and vegetables, indicating that the promotion of an increased dietary intake of fruits and vegetables and dairy is advisable in this chronically disabled group of individuals.

There are two forms of fat-soluble vitamin K, vitamin K1 or phylloquinone and vitamin K2 or menaquinones. Dietary sources of vitamin K1 or phylloquinone are generally supplied through vegetables, especially green leafy vegetables, vegetable oils, and some fruits. Meat, dairy, and eggs have a lower content and are a modest source of vitamins K1 and K2, respectively. In addition, not only do fermented foods have high levels of menaquinones, but the gastrointestinal tract bacteria also produce menaquinones. The most striking difference between vitamin K groups in our study was the consumption of fruits and vegetables, with the majority of the sample (e.g., BEL vitamin K group) having less than three servings of fruit and vegetables combined per day. In the stroke population of this study, the consumption of meat did not appear to influence vitamin K levels, perhaps because both groups on average had an adequate intake of this protein source. However, milk intake was associated with vitamin K levels, providing some evidence that increased dairy intake may be another option to increase vitamin K levels, especially as vitamin D levels were below recommendations across this group of stroke survivors.

As a group, absolute and relative amounts of protein intake fall within dietary guidelines for healthy adults despite the significant muscle atrophy that we have previously observed in this population [18,19]. In addition, absolute and relative carbohydrate intake and fat intake are similar to the national trends but slightly above (35%) what is recommended for fat intake (<30% fat). In this group, sodium intake is high, which emphasizes the need for a better adherence to lowering salty foods in the diet and eliminating the addition of salt in meals. Although our sample had a limited number of women, women had low intakes of calcium, vitamin D, E and vitamin A, suggesting the need for calcium+vitamin D supplements in this patient population.

Vitamin K is an essential protein in blood clotting and osteogenesis. It has a role in the prevention of vascular calcification and the acceleration of atherosclerosis. The potential relationship between vitamin K intake and cardiometabolic disease [3,4] introduces vitamin K intake as a conceivable additional measure when assessing chronic stroke survivors’ risk of recurrence. Stroke recurrence occurs at varying rates of 7–20% at 1 year and 16–35% at 5 years post stroke [20,21]. A recent review by Khanevski and colleagues, looking at predictors of ischemic stroke recurrence, determined that patients with lower modified Rankin Scale (mRS) scores at discharge, a more frequent history of previous ischemic stroke (IS), transient ischemic attack (TIA), or infarcts seen on magnetic resonance imaging (MRI), a history of hypertension, and a more frequent prescription of antiplatelet and antihypertensive drugs, were more likely to experience recurrence [20]. Other works identified diabetes mellitus as a factor associated with increased recurrence as well [21,22]. More information is needed to better understand whether vitamin K intake could be used as an easy predictive marker for a secondary stroke. Due to vitamin K’s potential therapeutic interactions with various diseases, controlled supplementation may be indicated for individuals struggling to consume adequate amounts. Vitamin K supplementation should be considered as a potential adjuvant therapy to address atherosclerosis. Patients with cardiovascular disease risk factors and a desire to improve vascular condition should first discuss the benefits and risks of vitamin K supplementation with their medical care provider. These discussions are most relevant in patients on anticoagulants, such as warfarin or coumadin [10].

Many older individuals are recommended to take multivitamin supplements in order to meet DRI levels. Yet, in our group, there was a low number of participants (<20%) who were on multivitamin supplements. Vitamin K, along with vitamin A, C, E and B complexes and minerals such as iron and zinc are commonly included in multivitamin supplements. Because the diet analysis program does not account for vitamins and minerals from a multivitamin, the amount of vitamin K and other nutrients may be underestimated in these individuals.

There is some rationale to investigating medication use and its interaction with vitamin K. In our sample, approximately half of the participants taking anticoagulants (three out of five) were taking warfarin/coumadin. The current recommendations are that these individuals are counselled not to eat green leafy vegetables or are counselled to consume an appropriate amount of vitamin K which could lead to lower vitamin K intake. This advice could be modified a bit with proper patient education and nutritional counseling to maintain constant vitamin K intake. Preliminary research from the American Society of Nutrition [9] suggests that the intake of green leafy vegetables can be added to the diet with a consistent dietary modification and to adjust the warfarin/coumadin dose based on the INR. This newer modification does not apply in the presence of an unstable INR level. However, given that, in our study, only a very small number of participants out of the whole sample (5/60) were on anticoagulants, our findings of low vitamin K intake across this group of stroke survivors is unlikely to be due to medication use, per se. Furthermore, given that the newer agents for anticoagulation, rivaroxaban and apixaban, do not have the same dietary restrictions, this will inherently not be a confounding factor. The investigation of medication use is further warranted given the increased risk of osteoporotic fracture seen in elderly patients when receiving long-term treatment with vitamin K antagonists [23]. For this reason, discussions on the choice of anticoagulant should include screening for osteoporotic fracture risk and other prescribed medications.

In addition to the lack of accounting for multivitamin use in the nutrition analysis software, other study limitations include variations in the length of the food record, the lack of a comparison group, the lack of measurements of serum markers of vitamin K, the lack of classifications of different categories of vegetables (e.g., green leafy vegetables), and the small sample size of women. Because we did not account for various factors related to vitamin K uptake (e.g., genetic profile, nutrient-nutrient interactions), conclusions drawn from this study should be carefully interpreted. However, detailed medical history, functional assessments, and the measurement of body composition provide a careful characterization of study participants. Furthermore, vegetable intake, albeit only green leafy vegetables, has been shown to be associated with serum markers of vitamin K [24]. Future research could investigate how a dietary intervention program, ideally led by a Registered Dietitian, impacts the intake of vitamin K in this population or whether a genetic predisposition (pharmacogenetics), such as polymorphisms of VKCOR1 and CYP2C9, plays a role in these relationships.

In conclusion, the inadequate intake of fruits and vegetables in the study population is likely a driving factor for the low intake of vitamin K reported in the participants’ food records. Given that the majority of this study population did not reach the DRI for vitamin K, it is advisable to promote the adequate intake of food rich in vitamin K. Further work is needed to determine the significance of the low vitamin K intake in this population.

## Figures and Tables

**Table 1:** Demographics.

	Group (*n* = 60)	Men (*n* = 49)	Women (*n* = 11)
Age, years	61.7 ± 7.2	62.5 ± 7.7	59.5 ± 4.7
Ethnicity, white/black	25/34	21/28	5/6
Body Weight, kg	91.13 ± 19.48	95.78 ± 18.38	77.78 ± 15.65
BMI, kg/m^2^	30.36 ± 6.61	31.35 ± 6.57	28.34 ± 6.45
Body Fat, %	35.32 ± 7.76	34.29 ± 7.87	39.25 ± 6.15
Fat mass, kg	32.51 ± 12.09	33.95 ± 12.29	31.04 ± 11.31
Lean mass, kg	54.86 ± 10.71	58.18 ± 9.23	43.98 ± 7.21
Physical Function			
6-min walk, ft	977 ± 401	977 ± 397	978 ± 434
10MWU, m/s	1.07 ± 0.51	1.03 ± 0.43	1.26 ± 0.80
10MWF, m/s	1.54 ± 1.21	1.53 ± 1.29	1.59 ± 0.78
Grip strength, kg			
Non-Paretic	34.02 ± 11.31	37.46 ± 9.38	19.89 ± 6.64
Paretic	19.96 ± 14.64	21.68 ± 15.65	12.89 ± 5.86
Medication, *n* (%)			
Antiplatelet	40 (67)	33 (67)	7 (63)
Anticoagulant	5 (8)	5 (10)	0 (0)
Statins	38 (63)	32 (65)	6 (55)
Insulin	4 (7)	2 (4)	1 (9)
Diabetes medication	20 (33)	16 (33)	4 (36)
Diuretic	17 (28)	16 (33)	1 (9)

Values are expressed as means ± standard deviation (10MWU = 10-m walk usual pace; 10MWF = 10-m walk fast pace).

**Table 2:** Dietary intake.

	Group (*n* = 60)	Men (*n* = 49)	Women (*n* = 11)
Energy, kcal	1762 ± 621	1790 ± 637	1634 ± 557
Protein, g	70 ± 20	71 ± 21	65 ± 16
Kcal from Protein, %	17.1 ± 4.8	17.1 ± 4.9	17.2 ± 4.5
carbohydrate, g	207 ± 85	212 ± 89	184 ± 60
Kcal from carbohydrate, %	47.0 ± 8.3	46.9 ± 8.1	47.8 ± 9.6
Fat, g	68 ± 30	71 ± 32	59 ± 16
Kcal from Fat, %	35.4 ± 6.6	35.7 ± 6.9	34.1 ± 5.3
Sodium, mg	2980 ± 1535	3158 ± 1615	2191 ± 732
Potassium, mg	2186 ± 2132	2285 ± 2324	1739 ± 795
Vitamin K, mcg	98 ± 150	100 ± 162	89 ± 81
Vitamin D, mcg	3.70 ± 3.4	4.1 ± 3.5	2.0 ± 1.9
Calcium, mg	664 ± 346	684 ± 361	571 ± 260
Vitamin C, mg	73 ± 57	71 ± 58	84 ± 54
Vitamin A, mcg RAE	661 ± 1207	736 ± 1352	387 ± 191
Iron, mg	12.9 ± 5.9	13.5 ± 6.2	10.1 ± 3.0
Vitamin E, mg	1.7 ± 4.0	1.7 ± 4.2	1.8 ± 2.7
Servings starch, *n*	6.63 ± 3.10	6.93 ± 3.27	5.27 ± 1.72
Servings fruit, *n*	1.81 ± 1.34	1.76 ± 1.27	2.00 ± 1.61
Servings vegetables, *n*	1.98 ± 2.53	1.95 ± 2.69	2.09 ± 1.71
Servings fat, *n*	5.70 ± 3.49	5.75 ± 3.69	5.50 ± 2.54
Servings high fat meat, *n*	1.20 ± 0.91	1.30 ± 0.96	0.75 ± 0.49
Servings medium fat meat, *n*	2.14 ± 1.81	2.23 ± 1.88	1.77 ± 1.51
Servings lean meat, *n*	1.54 ± 1.30	1.74 ± 1.36	0.91 ± 0.83
Servings very lean meat, *n*	1.92 ± 1.59	2.05 ± 1.57	1.55 ± 1.67
Servings whole milk, *n*	0.50 ± 0.63	0.61 ± 0.65	0.00 ± 0.00
Servings low fat milk, *n*	0.40 ± 0.49	0.37 ± 0.44	0.75 ± 1.06
Servings skim milk, *n*	0.45 ± 0.64	0.45 ± 0.80	0.44 ± 0.44
Servings other Carbohydrate, *n*	3.31 ± 2.79	3.52 ± 2.89	2.45 ± 2.45

Values are expressed as means ± standard deviation. RAE = retinol activity equivalent.

**Table 3:** Macronutrient and food group exchanges.

	Below Vitamin K DRI (*n* = 49)	Met Vitamin K DRI (*n* = 11)	*p*-Value
Energy, kcal	1769 ± 657	1729 ± 455	0.850
Protein, g	69 ± 20	72 ± 19	0.763
Kcal from Protein, %	17.1 ± 4.8	17.2 ± 5.2	0.976
Carbohydrate, g	204 ± 86	216 ± 86	0.672
Kcal from carbohydrate, %	46.9 ± 7.5	47.9 ± 12.1	0.720
Fat, g	69 ± 30	67 ± 30	0.127
Kcal from Fat, %	35.6 ± 5.1	34.5 ± 11.8	0.488
Servings starch, *n*	6.92 ± 2.90	5.32 ± 3.76	0.123
Servings fruit, *n*	1.68 ± 1.17	2.39 ± 1.88	0.106
Servings vegetables, *n*	1.43 ± 1.32	4.36 ± 4.59	0.000
Servings fat, *n*	5.47 ± 3.53	6.73 ± 3.26	0.284
Servings high fat meat, *n*	1.26 ± 0.94	0.79 ± 0.57	0.202
Servings medium fat meat, *n*	2.04 ± 1.77	2.67 ± 2.06	0.346
Servings lean meat, *n*	1.62 ± 1.38	1.14 ± 0.57	0.381
Servings very lean meat, *n*	1.74 ± 1.53	2.79 ± 1.70	0.114
Servings whole milk, *n*	0.50 ± 0.63	0	N/A
Servings low fat milk, *n*	0.40 ± 0.49	0	N/A
Servings skim milk, *n*	0.19 ± 0.33	1.00 ± 0.84	0.007
Servings other carbohydrate, *n*	3.16 ± 2.70	4.06 ± 3.27	0.385

Values are means ± standard deviation. *p* < 0.05 was considered significant. DRI= Dietary Reference Intake.

**Table 4:** Micronutrient and Dietary Reference Intake (DRI) intake.

	Daily Recommended Intake (DRI)	Below Vitamin K Recommendation (*n* = 49)	Met Vitamin K Recommendation (*n* = 11)	*p*-Value
Vitamin K, mcg	*Men:* 120 *Women:* 90	45 ± 44	330 ± 225	0.000
Vitamin D, mcg	*Both sexes 51–70 years:* 15	3.4 ± 3.4	4.9 ± 3.1	0.212
Calcium, mg	*Men 51–70 years:* 1000 *Women 51–70 years:* 1200	602 ± 322	940 ± 321	0.003
Vitamin C, mg	*Men:* 90 *Women:* 75	69 ± 57	89 ± 55	0.298
Vitamin A, mcg RAE	*Men:* 900 *Women:* 700	436 ± 352	1613 ± 2559	0.007
Vitamin E, mg	*Box sexes:* 15	1.0 ± 1.4	4.6 ± 8.3	0.005
Iron, mg	*Box sexes:* 8	12.6 ± 5.8	14.1 ± 6.5	0.437
Sodium, mg	*Both sexes:* 1500	3104 ± 1619	2431 ± 954	0.191
Potassium, mg	*Men:* 3400 *Women:* 2600	2071 ± 2262	2692 ± 1383	0.387

Values are means ± standard deviation. *p* < 0.05 was considered significant. RAE = retinol activity equivalent.

**Table 5:** Reported supplementation.

Dietary Supplement	Group (*n* = 60)	Below DRI (*n* = 49)	Met DRI (*n* = 11)
No MVI, *n* (%)	49 (82)	Vitamin K: 38 (78) Vitamin A: 27 (55) Vitamin E: 46 (94)	Vitamin K: 11 (100) Vitamin A: 9 (82) Vitamin E: 1 (9)
No Vitamin D supplement, *n* (%)	41 (68)	41 (84)	0 (0)
No Ca supplement, *n* (%)	55 (92)	48 (98)	7 (64)

MVI—multivitamin.
